# Psychological Impact of Working in Intensive Care on Nurses

**DOI:** 10.1192/j.eurpsy.2026.11242

**Published:** 2026-07-31

**Authors:** A. Aloui, I. Kacem, N. Ganoun, A. chouchane, M. Bouhoula, M. Makhloufi, H. Kalboussi, O. El Maalel, S. Chatti

**Affiliations:** Department of Occupational Medicine, Farhat Hached University Hospital, Sousse, Tunisia

## Abstract

**Introduction:**

Intensive care units provide critical care to severely ill patients, requiring complex monitoring and interventions performed by highly skilled medical teams. Nurses in these units are exposed to high levels of stress and emotional burden, placing their mental health at risk. This study explores the psychological impact on nurses working in intensive care.

**Objectives:**

To describe the psychological experience of nurses in intensive care units and its impact on their mental health.

**Methods:**

This is a descriptive quantitative study using a pre-designed questionnaire administered to 50 nurses working in intensive care units during January, February, and March 2025.

**Results:**

The study population was predominantly female (66%) with a sex ratio of 0.51. More than one-third of nurses (38%) were assigned to the regional hospital of Bizerte, and 64% worked in shifts. Over two-thirds of respondents (68%) had less than five years of professional experience in the unit, with a mean of 4.6 ± 2.8 years. Half of the respondents (50%) reported high work pressure in intensive care, while 42% indicated that they faced weekly challenges beyond their current competencies. The majority (86%) reported experiencing professional burnout. More than two-thirds (71%) identified increased stress as the main change in mood or emotional state while working in intensive care. Furthermore, 86% of participants supported the implementation of continuing education sessions focused on psychological skills to better cope with the emotional challenges associated with intensive care.

**Conclusions:**

The study highlights the urgent need to support the mental health of nurses in intensive care units by implementing targeted interventions to improve their well-being and the quality of patient care.

**Disclosure of Interest:**

None Declared

